# Cytokine signatures in chronic fatigue syndrome patients: a Case Control Study and the effect of anakinra treatment

**DOI:** 10.1186/s12967-017-1371-9

**Published:** 2017-12-29

**Authors:** Megan E. Roerink, Hans Knoop, Ewald M. Bronkhorst, Henk A. Mouthaan, Luuk J. A. C. Hawinkels, Leo A. B. Joosten, Jos W. M. van der Meer

**Affiliations:** 1 Department of Internal Medicine, Radboud University Medical Center, Nijmegen, The Netherlands; 2 Department of Medical Psychology, Academic Medical Center (AMC), University of Amsterdam, Amsterdam, The Netherlands; 3 Department for Health Evidence, Radboud University Medical Center, Nijmegen, The Netherlands; 4 Olink Proteomics, Uppsala, Sweden; 5 Department of Gastroenterology-Hepatology, Leiden University Medical Center, Leiden, The Netherlands

**Keywords:** Chronic fatigue syndrome, Proximity extension assay, Anakinra, IL-12p40, CSF-1

## Abstract

**Background:**

Cytokine disturbances have been suggested to be associated with the Chronic Fatigue Syndrome/Myalgic encephalomyelitis (CFS/ME) for decades.

**Methods:**

Fifty female CFS patients were included in a study on the effect of the interleukin-1-receptor antagonist anakinra or placebo during 4 weeks. EDTA plasma was collected from patients before and directly after treatment. At baseline, plasma samples were collected at the same time from 48 healthy, age-matched female neighborhood controls. A panel of 92 inflammatory markers was determined in parallel in 1 μL samples using a ‘proximity extension assay’ (PEA) based immunoassay. Since Transforming growth factor beta (TGF-β) and interleukin-1 receptor antagonist (IL-1Ra) were not included in this platform, these cytokines were measured with ELISA.

**Results:**

In CFS/ME patients, the ‘normalized protein expression’ value of IL-12p40 and CSF-1 was significantly higher (p value 0.0042 and 0.049, respectively). Furthermore, using LASSO regression, a combination of 47 markers yielded a prediction model with a corrected AUC of 0.73. After correction for multiple testing, anakinra had no effect on circulating cytokines. TGF-β did not differ between patients and controls.

**Conclusions:**

In conclusion, this study demonstrated increased IL-12p40 and CSF-1 concentrations in CFS/ME patients in addition to a set of predictive biomarkers. There was no effect of anakinra on circulating cytokines other than IL-1Ra.

*Trial Registration*: ClinicalTrials.gov Identifier: NCT02108210, Registered April 2014

**Electronic supplementary material:**

The online version of this article (10.1186/s12967-017-1371-9) contains supplementary material, which is available to authorized users.

## Background

Chronic fatigue syndrome/Myalgic encephalomyelitis (CFS/ME) is a condition of unknown origin that is characterized by severe fatigue for more than 6 months leading to significant disability. To fulfill the CFS/ME criteria as recommended by the Centers for Disease control (CDC), patients also have to report at least four out of eight of the accompanying symptoms (e.g. muscle pain, post-exertional fatigue, headache, etc.) [1, 2]. With CFS/ME being an exclusionary diagnosis, patients often report to have symptoms for several years before being diagnosed [3]. Most of the current case definitions suggest a collection of mandatory diagnostics to exclude common causes for fatigue such as anemia and thyroid illnesses [1, 4], but there is a need for more specific tests to diagnose patients. Another advantage of such a test is that it might be easier to define CFS/ME subgroups [5], for example those patients that would or would not respond to an immune intervention. Last but not least, a distinctive marker or set of markers may point to relevant pathogenetic mechanisms that can be further explored.

In the past years, numerous studies have been performed searching for potential biomarkers [6]. Because of the resemblance of CFS/ME with symptoms that characterize immune activation, there has been a particular interest in the immune system with studies measuring lymphocyte subsets [7, 8], cytokine production [9–11], and single nucleotide polymorphisms in immune related genes [12, 13]. However, despite a large number of studies conducted, this has not led to a unified conclusion useful for clinical practice. Studies are largely contradicting, and a recent systematic review on circulating cytokines did not find evidence for altered cytokine concentrations in CFS/ME, with the exception of transforming growth factor-beta (TGF-β) [14]. TGF-β appeared to be elevated in 63% of the selected studies. This was also found in a recent study on cytokine signatures in CFS [15]. Other cytokines were only elevated in a minority of studies, for example interleukin-1α (IL-1α) in 27% of the studies, interleukin 12 (IL-12) in 18%, interleukin 23 (IL-23) 25%, and interleukin 8 (IL-8) in 29% of studies. Some studies only found differences when differentiating between patients with long and short illness duration [9, 16].

In order to make progress on the role of the immune system in CFS/ME, we have to critically review the studies that have been performed, and try to clarify the reasons for these discrepancies. When measuring circulating cytokines, several issues have to be taken into account. First, patient selection is important. Studies often combine different cohorts of patients, recruit employees as controls, or controls who participated in previous studies [9, 17], and this may lead to different pre-analytical procedures. The latter is especially an important issue in this context. Cytokines may be released ex vivo by different circulating cells, and collection tubes, storage time, number of freeze–thaw cycles, and processing protocols have been found to be of major influence [18, 19]. To make a reliable comparison between patients and controls, especially in CFS/ME where circulating cytokines are expected to be low, it is essential that the pre-analytical process in these groups is identical.

Another important issue is the type of analysis used to determine cytokine concentrations. Most studies measuring cytokines use antibody based enzyme-linked immuno sorbent assays (ELISA) [14]. However, limitations of this technique are that multiplex forms of the assays often use only one antigen-binding antibody to detect the protein, which limits detection specificity as well as sensitivity [20]. Sandwich ELISA achieves better performance by using pairs of antibodies for each targeted protein, but the assays typically need relatively large sample volumes for analyses of single protein species, limiting throughput and spending precious samples. By contrast, the proximity extension assay (PEA) uses dual antibody recognition with oligonucleotide-conjugated antibodies in multiplex assays with modest requirements for sample volumes [20, 21]. Upon simultaneous binding of the correct pair of antibodies, their attached oligonucleotides anneal to each other and can be enzymatically extended, forming specific DNA sequences that can be quantified using quantitative real-time polymerase chain reactions (qPCR).

In this study, cytokine profiles of female CFS/ME patients participating in a randomized controlled trial on the effect of IL-1 inhibition on fatigue severity [22] were compared with age- and gender-matched healthy neighborhood controls. The rationale of this RCT was that IL-1—despite the fact that it is notoriously difficult to measure in the circulation—may play a pathophysiological role in CFS, and its activity may be confined to the brain compartment [23]. In addition to the cytokines included in the PEA platform, TGF-β and the IL-1 receptor antagonist (IL1-Ra), were measured separately using an ELISA. Pre-analytical procedures were identical for patients and controls. Furthermore, the effect of IL-1 inhibition using the IL-1-receptor antagonist anakinra for 1 month on circulating cytokine concentrations was assessed. As reported in detail elsewhere, the study did not demonstrate a beneficial therapeutic effect in these patients [24].

## Methods

### Patients and design

All patients participated in a double-blind randomized controlled trial (RCT) on the effect of IL-1 inhibition on CFS/ME-related symptoms, of which the results were reported elsewhere [24]. The study was conducted at the Department of Internal Medicine and Expert Center for Chronic Fatigue (ECCF) of the RadboudUMC, Nijmegen, the Netherlands. Details of the study were described previously [22]. In short, fifty female patients between 18 and 59 years old were included when they fulfilled the CDC criteria for CFS/ME [1, 2]. As recommended by the CDC criteria, patients can only be included when the body mass index (BMI) is ≤ 40 kg/m^2^. Main exclusion criteria were the presence of a somatic disease that could explain severe fatigue (sleep apnea, anemia, etc.), psychiatric comorbidity (e.g. depression, anxiety disorders) or the use of medication (with the exception of oral contraceptives and paracetamol). Patients were asked to bring a healthy, female, neighborhood control, without complaints of fatigue and within the same age range (± 5 years), to their first study visit.

After inclusion, patients were randomized 1:1 to either daily subcutaneous (s.c.) injections with anakinra (100 mg/day) or placebo (mixture of sodium citrate, sodium chloride, and polysorbate) for a duration of four weeks. Controls did not receive an intervention. Anakinra and placebo were provided by the Swedish Orphan Biovitrum (Sobi, Stockholm, Sweden). The randomization list was computer-generated by the Department of Pharmacy [25]. Patients administered the study medication at home on a daily basis. Both the placebo and anakinra syringes had an identical appearance, and drug adherence was evaluated as described previously [22, 24].

All participants provided written and oral informed consent before inclusion. The hospitals’ ethics committee approved the study protocol (2014/025). The study was performed in accordance with the declaration of Helsinki.

### Questionnaires

Fatigue was measured in both patients and controls using the fatigue severity subscale of the checklist individual strength (CIS), which has been used frequently in CFS/ME patients [26, 27]. Scores on the CIS-f can vary between 8 and 56, and a score ≥ 35 reflects severe fatigue [28]. Psychological distress was measured with the total score on the Symptom Checklist-90 (SCL-90) [29].

### Peripheral blood collection

Morning blood samples were collected from all patients prior to the first s.c. injection, and after 4 weeks of treatment. Samples of controls were collected and processed simultaneously with those of patients before treatment. There were no specific instructions with respect to food intake prior to blood sampling. Venous blood was collected in EDTA tubes, and kept on ice until centrifugation, which was performed within 2–3 h. Next, samples were centrifuged at 2960×*g* for 10 min at 4 °C. Plasma aliquots were then frozen at − 80 °C for a maximal duration of 2 years. Analyses for all patients and controls were run at the same time.

### PEA assay

Inflammation biomarker profiles were analyzed by the analysis service of Olink Proteomics AB (Uppsala, Sweden), using their PEA based Proseek© Multiplex Inflammation panel^96*96^ [21, 29, 30]. This analysis simultaneously measures 92 selected inflammatory proteins, listed in Additional file 1, using only 1 μL of plasma. For each protein, there are two separate antibodies connected to one oligonucleotide each. After binding by the antibody pair to its target, the 3′ ends of the oligonucleotides hybridize, priming a DNA polymerization reaction that forms a protein-specific reporter DNA-sequence for each detected protein molecule. The reporter DNA strands are then quantified using qPCR. Four internal controls and two external controls were included in each assay. The raw Cq values were normalized for variation between and within runs and converted into Normalized Protein Expression Units (NPX). The NPX values are expressed on a Log2 scale where one unit higher NPX values represent a doubling of the measured protein concentrations. This arbitrary unit can be used for relative quantification of proteins and comparing the fold changes between groups.

Based on the CFS/ME literature, 20 cytokines were selected to be of special interest; CD40L (CD40 ligand), CXCL-9 (chemokine ligand 9), CXCL-10 (chemokine ligand 10), CCL-2 (MCP-1), CCL-11 (eotaxin), IFN-γ (interferon gamma), IL-1α (interleukin-1 alpha), IL-2 (interleukin-2), IL-4 (interleukin-4), IL-6 (interleukin-6), IL-7 (interleukin-7), IL-8 (interleukin-8), IL-10 (interleukin-10), IL-12p40 (interleukin-12 subunit p40), IL-17A (interleukin-17A), CSF-1 (macrophage colony-stimulating factor 1), TNF-β (tumor necrosis factor-beta), TRAIL (TNF-related apoptosis-inducing ligand), TGF-α (transforming growth factor alpha), and TNF (tumor necrosis factor) [9, 14, 31].

### Elisa

Total TGF-β1 levels were measured by sandwich ELISA as described in detail previously (R&D systems) [32]. All samples were acid activated to activate latent TGF-β (1 M hydrochloric acid, 30 min, room temperature). Analysis was performed at the Leiden University Medical Center. IL-1Ra ELISA (R&D systems) was performed at the Radboud University Medical Center.

### Statistical analysis

Study data were analyzed using IBM SPSS statistic package version 22 and *R* [33]. All continuous variables are presented as means and standard deviations (SD) or medians and ranges, and categorical variables as percentages.

Inflammatory markers were excluded if > 25% of the measurements were below the detection limit. Remaining missing values were imputed with a random value between 0 and the LOD for the protein at hand, a method that avoids the artificial reduction of the standard deviation that is a consequence of imputing the values LOD/2 or LOD/√2. For the baseline comparison of twenty pre-selected cytokines, analysis of covariance (ANCOVA) was performed with age and BMI added as covariates. Based on the result of a previous study, the same analysis was repeated dividing the patient group into patients with a long illness duration (> 3 years) and patients with a short illness duration [9].

In order to establish a predictive model, a logistic regression model was selected using the LASSO regression strategy that aims at eliminating predictors with only marginal predictive performance [34]. As potential predictors for CFS/ME, the cytokine concentrations supplemented with age and BMI were used. To determine the performance of this model, the area under the ROC-curve (AUC) was calculated [35]. As the model is evaluated in the same population that is used for construction, the predictive performance will be overestimated. To correct for this optimism, new populations were generated using bootstrap sampling. In each population the same modeling strategy was used. Each prediction model was then evaluated in both the bootstrap population and the original population. After 500 repetitions of this process, the differences between performance in bootstrapped samples and the original population were used to estimate the optimism due to internal validation [36].

To determine the influence of IL-1Ra on cytokine concentrations, analysis ANCOVA was used with the cytokine concentration after 4 weeks as dependent variable, treatment as fixed factor, and concentration at baseline, age, and BMI as covariates.

## Results

### Patient characteristics

A total of 50 CFS/ME patients and 48 age-matched, neighborhood controls were included in the study. Two patients were not able to bring a healthy control at baseline. Table 1 displays demographic and fatigue-related characteristics. Within the CFS/ME group, there were 21 patients with a short illness duration (≤ 3 years, 58%) and 29 patients with a long illness duration (> 3 years, 42%). As expected, CFS/ME patients had a higher CIS-fatigue score than controls (52 ± 4 vs. 20 ± 11, p < 0.001). Total score on psychological distress was also significantly higher in patients (150 ± 30 vs 119 ± 32, p < 0.001). BMI, ethnicity, and percentage of patients using oral contraceptives did not significantly differ between groups.

**Table 1:** Baseline characteristics of chronic fatigue syndrome patients (CFS/ME) and healthy controls (HC)

	CFS/ME (n = 50)	HC (n = 48)	P value
Age, years	31 (10)	31 (10)	0.98
Ethnicity Caucasian (%) Other (%)	49 (98) 1 (2)	47 (98) 1 (2)	0.37
Body mass index, kg/m2	25.1 (4.5)	24.9 (4.4)	0.88
Oral contraceptives (%) Paracetamol (%)	22 (44) 22 (44)	16 (33) 8 (16)	0.28 0.002
Illness duration, months ≤ 3 years (%) > 3 years (%)	43 [7–109] 29 (58) 21 (42)	N/A	
Fatigue severity (CIS-fatigue)	52 (4)	20 (11)	< 0.001
Psychological distress (SCL-90)	150 (30)	119 (32)	< 0.001

Data are number (%), mean (SD), or median [range]

*CIS* checklist individual strength, *SCL-90* symptom checklist 90, *N/A* not applicable

### CFS/ME patients versus controls

**Fig. 1:**
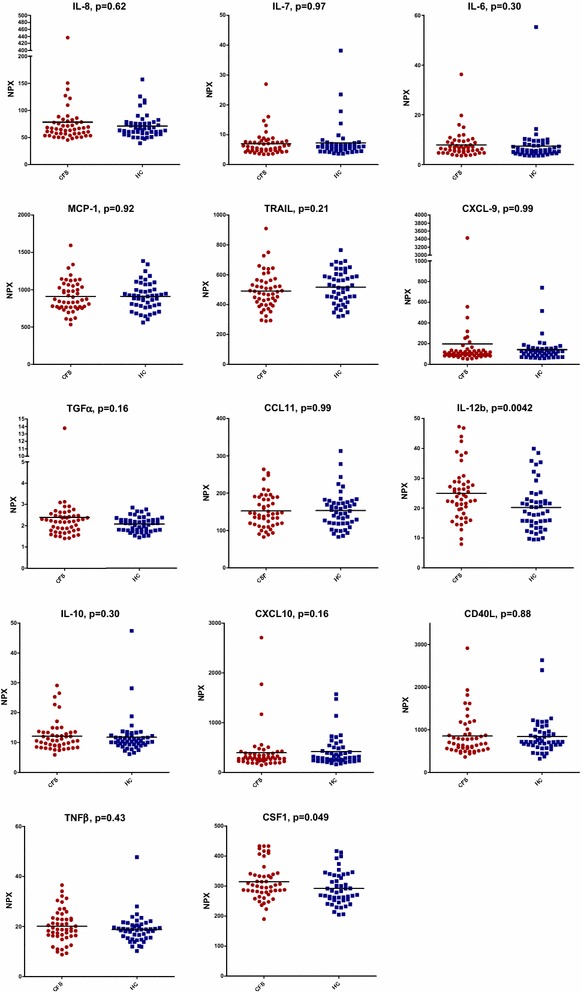
Normalized Protein Expression Units (NPX) values for patients with chronic fatigue syndrome (CFS/ME, n = 50) compared to healthy controls (HC, n = 48). Displays linear NPX values for patients and controls. P values were derived by analysis of covariance

Twenty pre-selected cytokines were compared between patients and controls, based on the recent CFS/ME literature [9, 14, 31]. For IFN-γ, IL-1α, IL-2, IL-4, IL17A, and TNF, more than 25% of samples was under the LOD in both patients and controls. Results for the remaining 14 cytokines are displayed in Fig. 1. In this exploratory analysis, both IL-12b and CSF-1 appeared to be elevated in CFS/ME patients (p value 0.004 and 0.049 respectively). Other cytokines did not differ between patients and controls. Dividing the patient group into those with short illness duration, and those with longer illness duration did not change these results (data not shown).

**Fig. 2:**
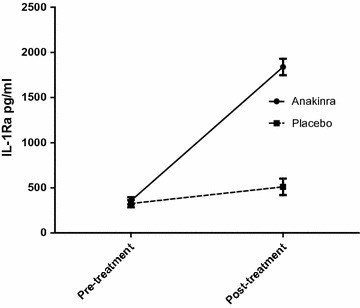
Concentration of IL-1Ra for patients treated with either anakinra or placebo. Displays IL-1Ra concentrations ± SEM in patients treated with either anakinra or placebo before and after treatment

There were no differences in TGF-β1 concentrations between patients and controls, as determined by ELISA (4520 ± 499 pg/mL versus 5972 ± 1279 pg/mL). IL-1Ra concentrations did not differ between patients and controls before anakinra treatment (Fig. 2).

### Prediction model

Of the 92 proteins measured, 22 appeared to be below the detection limit for > 25% of samples for both patients and controls (IFN-γ, IL-1α, IL-2, IL-4, IL17A, TNF, MCP-3, IL-17c, IL-20Ra, IL-2Rb, TSLP, IL-10Ra, IL-22Ra1, PD-L1, IL-24, IL-13, ARTN, IL-20, IL-33, LIF, NRTN, and IL-5). Although theoretically there is still information in the recorded concentrations for these cytokines, these proteins were excluded from the analysis as they were considered not to be candidates with a substantial predictive potential. The remaining 70 proteins were entered into the LASSO regression analysis in addition to age and BMI. Out of this total of 72 variables entered, 47 appeared in the final regression model (Table 2). 22 variables had a positive association with the risk of being a CFS/ME patient, and a negative association was present for 23 variables. To determine the performance of this model, an AUC was calculated with correction for optimism. Optimism in the current model was 0.265, which resulted in a corrected AUC value of 0.734.

**Table 2:** LASSO regression analysis: factors associated with the risk of being a CFS/ME patient versus healthy controls

Protiens that are positively associated with the risk of being a CFS/ME patient:		Proteins that are negatively associated with the risk of being a CFS/ME patient:	
Protein	Weight	Protein	Weight
TWEAK	5077	BetaNGF	− 6683
CCL4	4182	LIFR	− 4053
IL12B	3898	HGF	− 2153
CDCP1	3611	CXCL6	− 1849
VEGFA	3138	4EBP1	− 1317
CSF1	2810	SCF	− 1307
IL10RB	1737	MMP1	− 1256
CCL11	1354	ADA	− 1237
CD5	1087	CXCL10	− 1223
MCP1	1047	IL18R1	− 1076
CASP8	0865	CXCL9	− 0697
FGF5	0758	CCL28	− 0611
IL6	0737	CCL25	− 0557
CCL23	0591	OSM	− 0544
CX3CL1	0568	CCL20	− 0514
ST1A1	0307	CCL19	− 0507
TNFSF14	0303	TRANCE	− 0498
CD244	0302	NT3	− 0489
IL10	0287	MCP4	− 0458
CXCL5	0245	TRAIL	− 0406
LAPTGFbeta1	0166	ENRAGE	− 0356
OPG	0107	CD6	− 0109
		TNFB	− 0062
		MMP10	− 0033
		FGF23	− 0017
Optimism: 0.2652 AUC: 0.9996 Corrected AUC: 0.7344			

Given associations are all conditional on the other predictors

### Influence of IL-1Ra on circulating cytokines

**Fig. 3:**
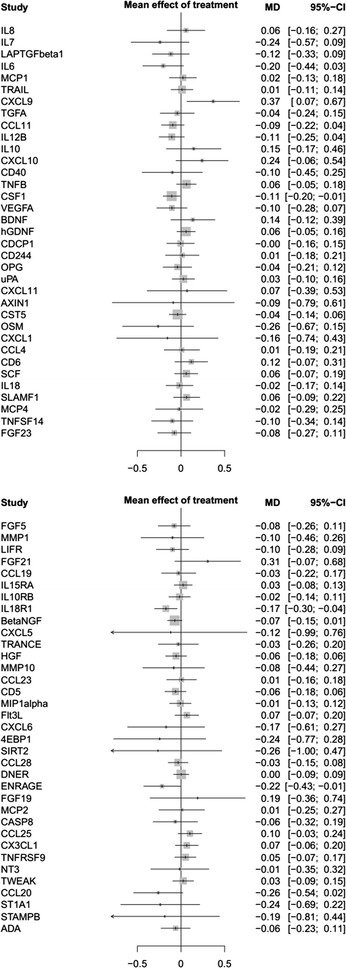
Treatment effect of the 4 week intervention period on circulating cytokines. Displays the difference and 95% confidence intervals between study arms for protein values after treatment (anakinra:controls)

In accordance with the analysis of patients versus controls, in 22 cytokines the NPX value was below the detection limit in more than 25% of cases. These cytokines were excluded from the analysis. One patient in the anakinra group discontinued treatment after 2 weeks as a consequence of an adverse event and was excluded from the analysis. IL-1α, a cytokine of special interest, was not detectable in more than 75% of samples both before and after treatment. In Fig. 3 the influence of anakinra vs placebo is displayed for all detectable cytokines with corresponding 95% confidence intervals (95% CI). In the anakinra group there appeared to be an inhibiting effect of anakinra on CSF-1, IL-18R1, and ENRAGE. In addition there was a stimulating effect on CXCL-9; for the remaining variables there was no influence of anakinra. As expected, IL-1Ra was significantly higher in those patients treated with anakinra (p < 0.001, Fig. 2). Of the 25 patients treated with anakinra, 23 patients had a concentration above the detection limit of 2000 pg/mL.

## Discussion

In this study differences in plasma cytokine profiles of 50 CFS/ME patients compared to 48 healthy age-matched neighborhood controls were evaluated using a PEA protein assay. The concentration of IL-12p40 and CSF-1 were significantly higher in CFS/ME patients. For the remaining cytokines of interest based on the current CFS/ME literature, no differences could be found comparing them individually. However, using the complete inflammatory profile, patients and controls could be discriminated using 22 variables with a positive association and 24 variables with a negative association.

IL-12b, also known as IL-12p40, was significantly higher in CFS/ME patients compared to healthy controls (p = 0.005). The IL-12p40 subunit is expressed by activated dendritic cells (DC) and combines with either subunit p35 or p19, to form IL-12 or IL-23 [37]. IL-12 targets T-cells and NK-cells, in which it induces IFN-γ production [38]. IL-23 has an important role in Th17 production, has an effect on memory T-cells, and appears to be critical in cerebral autoimmune inflammation [39]. Previous studies in CFS/ME patient also found increased concentration of both IL-12 [17, 40, 41], IL-12b [9] and IL23 [42]. A relationship between IL-12 and fatigue has been established in studies investigating the effect of administration of human recombinant IL-12 to treat ovarian cancer, and head and neck cancer [43, 44]. A proportion of treated patients developed fatigue, and combined with other side effects such as fever and chills, this toxicity had dose-limiting consequences. Furthermore, increased concentrations of IL-12 and IL-23 have especially been associated with multiple sclerosis, in addition to psoriasis, inflammatory bowel disease, cancer and rheumatoid arthritis [37]. However, in the light of the explorative nature of the current analysis, replication of this finding is important.

Increased concentrations of CSF-1 have not been previously been reported in CFS. CSF-1 or macrophage colony-stimulating factor (M-CSF) is a hematopoietic growth factor involved in proliferation and differentiation of monocytes and macrophages. Targeting CSF-1 has predominantly been described in cancer, where there appears to be an advantage when it is used in combination with other immune therapies [45]. Interestingly, the role of CSF-1 in the development of sickness behavior has recently been assessed by Müller et al. [46]. In this study, neutralization of the CSF-1 receptor prevented the development of sickness behavior in mice treated with an inflammatory stimulus. This behavioral response was mediated through increased IL-10 production. Increased IL-10 concentrations were also demonstrated in the hypothalamus, where the behavioral effect is most likely to be effected.

In contrast to the majority of previous studies measuring circulating cytokines, in this study there were no differences in TGF-β concentrations [14]. TGF-β influences cell proliferation, migration, and differentiation, and is known for its dual role in cancer [47]. TGF-β is released in large quantities by activated platelets [48]. Several factors, such as blood sampling procedures [49] and use of medication, influence the extent of platelet activation. A possible explanation for the increased TGF-β levels previously reported might be caused by differences in sample handling and drug use, which has recently been demonstrated [50]. In a recent CFS study, elevated TGF-β were also reported [15]. The method used by these investigators does not allow to assess the magnitude of the TGF-β concentrations, and there is little mention of potential confounder. For example, oral contraceptives induce platelet activation in humans [51], and directly cause increased TGF-β concentrations in rats [52].

These differences regarding sample handling might also be the explanation for the fact that previous studies found more cytokines to be differentially expressed in CFS/ME patients. A substantial proportion of studies included multiple cohorts in their analyses [9, 31], and although this gives the opportunity to investigate a larger number of patients, including patients coming from geographically different regions results in an inherent danger of inaccurate conclusions. This is because sample handling may have been different, for example use of different centrifuges, differences in ambient temperature, variations in collection time, and storage. Such differences in pre-analytic sample handling and storage, were part of the explanation for the spurious reports on the role of murine retroviruses in CFS/ME [53]. In addition, cytokine concentrations are known to be influenced by drug use. The use of non-steroidal anti-inflammatory drugs (NSAIDS), which are frequently used by CFS/ME patients, increase the production of various cytokines [54, 55]. Differences in use of NSAIDS and other medication between patients and controls, might have explained the reported increased levels of pro-inflammatory cytokines in previous studies.

Interestingly, combining all inflammatory markers yields a prediction model containing 47 markers with a corrected AUC of 0.73. To control for optimism, a bootstrapping method was used, which yielded a high correction factor of 0.265. Some of the measured proteins have a positive association with CFS/ME (IL-6, CSF-1), whereas for others there was a negative association (BetaNGF, CXCL-6). This selection of inflammatory markers could be a starting point for further studies investigating potential diagnostic markers in CFS/ME.

According to the 95% confidence intervals, there was an inhibiting effect of anakinra on circulating CSF-1, IL-18R1 and ENRAGE and a stimulating effect on CXCL-9. Given the large numbers of cytokines tested, these findings have to be interpreted with caution. It is important to mention that IL-1β was not included in the analysis, and IL-α was already below the detection limit before treatment in 82% of samples. It was expected that there would be a significant decrease of IL-6 in the anakinra group, as IL-1 induces IL-6 production, and is frequently used as a readout for IL-1 activity [56]. In previous studies, there was also a significant reduction of IL-6 concentrations after anakinra treatment. This was investigated in patients intravenously treated after stroke [57] and patients treated with subcutaneous injections for heart failure [58]. A possible explanation for the lack of a decrease in IL-6 concentrations is that in comparison to the situation in stroke patients where it has an important prognostic role [59], CFS/ME patients exhibit no increase of this cytokine at baseline. In stroke and heart failure an inflammatory response in which IL-1 plays a key role is now well established, but it is possible that IL-1 does not play a role in CFS. Since drug adherence was excellent, which is also reflected by the significant increase of IL-1Ra in the anakinra treated group, it is unlikely that lack of compliance is an explanation for this result.

This study has several strengths. To our knowledge, PEA-based assays have not previously been performed in CFS/ME patients. Over the past few years, there has been a search for sensitive methods to measure multiple inflammatory markers simultaneously in order to find potential biomarkers for CFS/ME. However, this is most commonly performed using multiplex bead-based immunoassays, that have limited sensitivity and specificity [20]. This is not the case for the PEA method, which has a much higher specificity as the signals can only be elicited by the cognate antibody pairs, while cross reactions between irrelevant antibody pairs are ignored [21]. Another asset of this study is the inclusion of neighborhood controls. Each patient was asked to bring a healthy, sex- and age-matched control, and blood withdrawal of both patient and control took place at the same time. Pre-analytic processes were identical, which was not the case in most of the previously published biomarker studies in CFS/ME [31, 41]. The third advantage of the current study is exclusion of patients who use medication, with the exception of oral contraceptives and paracetamol. CFS/ME patients frequently use a significant amount of medication, in a recent study 64% of patients used complementary and alternative medicine [60]. Another study found that > 90% of CFS/ME patients use at least one drug or supplement, especially antidepressants, sedatives and muscle relaxants [61].

A limitation of this study is the relatively small number of patients included measuring a large number of variables. However, this has been accounted for using the LASSO method for logistic regression, which is an elegant variable reduction method [62]. However, considering the large factor for optimism, the prediction model has to be interpreted with caution. Another limitation of the current study is the fact that IL-1β was not measured in plasma samples, although the value of this measurement is limited as IL-1β is usually undetectable in peripheral blood, even with the PEA method.

## Conclusion

In conclusion, this study demonstrated increased IL-12p40 and CSF-1 concentrations in CFS/ME patients in addition to a set of predictive biomarkers. There was no effect of anakinra on circulating cytokines other than IL-1Ra. As emphasized in this study, sample handling and diagnostic procedures are very important when measuring cytokines. Future studies should take this into account and in order to replicate findings, methods should be extensively reported.

## Additional file

**Additional file 1.** List of proteins included in the PEA analysis.

## Abbreviations

CFS/ME: chronic fatigue syndrome/Myalgic encephalomyelitis
PEA: proximity extension assay
CDC: centers for disease control
TGF-β: transforming growth factor-beta
IL-1α: interleukin-1α
IL-12: interleukin 12
IL-23: interleukin 23
IL-8: interleukin 8
ELISA: enzyme-linked immuno sorbent assay
qPCR: quantitative real-time polymerase chain reaction
IL-1Ra: IL-1 receptor antagonist
RCT: randomized controlled trial
ECCF: Expert Center for Chronic Fatigue
BMI: body mass index
s.c.: subcutaneous
CIS: checklist individual strength
NPX: Normalized Protein Expression Units
CD40L: CD40 ligand
CXCL-9: chemokine ligand 9
CXCL-10: chemokine ligand 10
CCL-2: MCP-1
CCL-11: eotaxin
ENRAGE: extracellular newly identified receptor for advanced glycation end-products binding protein
IFN-γ: interferon gamma
IL-1α: interleukin-1 alpha
IL-2: interleukin-2
IL-4: interleukin-4
IL-6: interleukin-6
IL-7: interleukin-7
IL-8: interleukin-8
IL-10: interleukin-10
IL-12p40: interleukin-12 subunit p40
IL-17A: interleukin-17A
CSF-1: macrophage colony-stimulating factor 1
TNF-β: tumor necrosis factor-beta
TRAIL: TNF-related apoptosis-inducing ligand
TRANCE: Tumor necrosis factor-related activation-induced cytokine
TGF-α: transforming growth factor alpha
TNF: tumor necrosis factor
TWEAK: tumor necrosis factor-like weak inducer of apoptosis
ANCOVA: analysis of covariance
AUC: area under the curve
NSAIDS: non-steroidal anti-inflammatory drugs
