# Supplementary material for: Cytokine signatures in chronic fatigue syndrome patients: a Case Control Study and the effect of anakinra treatment
Source: J Transl Med. 2017 Dec 29;15:267. doi: 10.1186/s12967-017-1371-9 (PMC5747240; doi:10.1186/s12967-017-1371-9)
Supplement: Supplementary file 1 — Additional file 1. List of proteins included in the PEA analysis. [file 12967_2017_1371_MOESM1_ESM.docx]

**S1** List of proteins included in the PEA analysis.

| **Full name** | **Abbreviation** |
| --- | --- |
| **Adenosine Deaminase** | ADA |
| **Artemin** | ARTN |
| **Axin-1** | AXIN1 |
| **Beta-nerve growth factor** | Beta-NGF |
| **Brain-derived neurothropic factor** | BDNF |
| **C-C motif chemokine 19** | CCL19 |
| **C-C motif chemokine 20** | CCL20 |
| **C-C motif chemokine 23** | CCL23 |
| **C-C motif chemokine 25** | CCL25 |
| **C-C motif chemokine 28** | CCL28 |
| **C-C motif chemokine 3** | CCL3 |
| **C-C motif chemokine 4** | CCL4 |
| **C-X-C motif chemokine 1** | CXCL1 |
| **C-X-C motif chemokine 10** | CXCL10 |
| **C-X-C motif chemokine 11** | CXCL11 |
| **C-X-C motif chemokine 5** | CXCL5 |
| **C-X-C motif chemokine 6** | CXCL6 |
| **C-X-C motif chemokine 9** | CXCL9 |
| **Caspase-8** | CAPS-8 |
| **CD40L receptor** | CD40 |
| **CUB domain-containing protein 1** | CDCP1 |
| **Cystatin D** | CST5 |
| **Delta and Notch-like epidermal growth factor-related receptor** | DNER |
| **Eotaxin** | CCL11 |
| **Eukaryotic translation initiation factor 4E-binding protein 1** | **4E-BP1** |
| **Fibroblast growth factor 19** | **FGF-19** |
| **Fibroblast growth factor 21** | **FGF-21** |
| **Fibroblast growth factor 23** | **FGF-23** |
| **Fibroblast growth factor 5** | **FGF-5** |
| **Fms-related tyrosine kinase 3 ligand** | **Flt3L** |
| **Fractalkine** | **CX3CL1** |
| **Glial cell line-derived neurotrophic factor** | **GDNF** |
| **Hepatocyte growth factor** | **HGF** |
| **Interferon gamma** | **IFN-gamma** |
| **Interleukin-1 alpha** | **IL-1 alpha** |
| **Interleukin-10** | **IL10** |
| **Interleukin-10 receptor subunit alpha** | **IL-10RA** |
| **Interleukin-10 receptor subunit beta** | **IL-10RB** |
| **Interleukin-12 subunit beta** | **IL-12B** |
| **Interleukin-13** | **IL-13** |
| **Interleukin-15 receptor subunit alpha** | **IL-15RA** |
| **Interleukin-17A** | **IL-17A** |
| **Interleukin-17C** | **IL-17C** |
| **Interleukin-18** | **IL-18** |
| **Interleukin-18 receptor 1** | **IL-18R1** |
| **Interleukin-2** | **IL-2** |
| **Interleukin-2 receptor subunit beta** | **IL-2RB** |
| **Interleukin-20** | **IL-20** |
| **Interleukin-20 receptor subunit alpha** | **IL-20RA** |
| **Interleukin-22 receptor subunit alpha-1** | **IL-22 RA1** |
| **Interleukin-24** | **IL-24** |
| **Interleukin-33** | **IL-33** |
| **Interleukin-4** | **IL-4** |
| **Interleukin-5** | **IL-5** |
| **Interleukin-6** | **IL-6** |
| **Interleukin-7** | **IL-7** |
| **Interleukin-8** | **IL-8** |
| **Latency-associated peptide transforming growth factor beta-1** | **LAP TGF-beta-1** |
| **Leukemia inhibitory factor** | **LIF** |
| **Leukemia inhibitory factor receptor** | **LIF-R** |
| **Macrophage colony-stimulating factor 1** | **CSF-1** |
| **Matrix metalloproteinase-1** | **MMP-1** |
| **Matrix metalloproteinase-10** | **MMP-10** |
| **Monocyte chemotactic protein 1** | **MCP-1** |
| **Monocyte chemotactic protein 2** | **MCP-2** |
| **Monocyte chemotactic protein 3** | **MCP-3** |
| **Monocyte chemotactic protein 4** | **MCP-4** |
| **Natural killer cell receptor 2B4** | **CD244** |
| **Neurotrophin-3** | **NT-3** |
| **Neurturin** | **NRTN** |
| **Oncostatin-M** | **OSM** |
| **Osteoprotegerin** | **OPG** |
| **Programmed cell death 1 ligand 1** | **PD-L1** |
| **Protein S100-A12** | **EN-RAGE** |
| **Signaling lymphocytic activation molecule** | **SLAMF1** |
| **SIR2-like protein 2** | **SIRT2** |
| **STAM-binding protein** | **STAMPB** |
| **Stem cell factor** | **SCF** |
| **Sulfotransferase 1A1** | **ST1A1** |
| **T cell surface glycoprotein CD6 isoform** | **CD6** |
| **T-cell surface glycoprotein CD5** | **CD5** |
| **Thymic stromal lymphopoietin** | **TSLP** |
| **TNF-beta** | **TNFB** |
| **TNF-related activation-induced cytokine** | **TRANCE** |
| **TNF-related apoptosis-inducing ligand** | **TRAIL** |
| **Transforming growth factor alpha** | **TGF-alpha** |
| **Tumor necrosis factor (Ligand) superfamily, member 12** | **TWEAK** |
| **Tumor necrosis factor** | **TNF** |
| **Tumor necrosis factor ligand superfamily member 14** | **TNFSF14** |
| **Tumor necrosis factor receptor superfamily member 9** | **TNFRSF9** |
| **Urokinase-type plasminogen activator** | **uPA** |
| **Vascular endothelial growth factor A** | **VEGF-A** |
